# Clinical Lectures on Diseases of the Eye

**Published:** 1866-04

**Authors:** E. L. Holmes

**Affiliations:** Lecturer on Diseases of the Eye in Rush Medical College, and Surgeon to the Chicago Charitable Eye and Ear Infirmary


### CHICAGO MEDICAL JOURNAL.

VOL. XXIII. APRIL, 1866. No. 4.

### ORIGINAL CONTRIBUTIONS.

*Clinical Lectures on Diseases of the Eye.* By E. L. HOLMES, M. D., Lecturer on Diseases of the Eye in Rush Medical College, and Surgeon to the Chicago Charitable Eye and Ear Infirmary.

### ASTHENOPIA.

GENTLEMEN,—The power of endurance, or, in popular language, the "strength" of the eye, is very different in different individuals, and not unfrequently in the same individual at different periods of life. While some persons can read, write, or engrave, for instance, many hours daily, and even at night, without fatigue, and are not disturbed by unsuitable light, or by motion, as in riding, others suffer fatigue and pain from the slightest effort to look upon near objects.

The class of cases to which I wish to call your attention to-day, is characterized, as you have already seen in several patients at the clinic, by inability to use the eyes upon near objects without pain in and around the eyes, temporary indistinctness of objects, with more or less congestion of the different tissues of the eye. To this condition of the eyes has been given the term Asthenopia—weak-sightedness. It should be remembered that this term signifies want of endurance, and not indistinctness of vision, as in incipient amaurosis.

In studying the phenomena peculiar to this condition, there are three principal elements to be investigated, which in fact divide these cases into three distinct classes.

The first class comprises those cases in which there is a pathological change in the ciliary muscle, not well understood, from which there is a debility or insufficiency of power, analogous to want of innervation in the general muscular apparatus of the body, in consequence of which exercise soon produces a sense of fatigue more or less painful.

The muscle cannot long remain in a state of contraction without a painful effort. It soon becomes wearied, and in spite of the patient it relaxes. In consequence of this, the convexity of the lens is decreased, and the focus of the eye is thrown behind the retina; objects become indistinct, the letters and lines for instance, in reading, "run together." At first there is a mere sense of weariness, but rest for a few moments, and looking at distant objects, restores to a certain degree the energy of the muscle; the sense of fatigue being relieved, the patient is able to use the eyes again for a short period, without inconvenience. Students, tailors, engravers and seamstresses are especially liable to this disease of the eyes. In the early stages of the affection, patients are able to labor many hours daily with only a feeling of weariness in the eyes at night, which is entirely relieved by sleep. After a period of time, however, such individuals experience fatigue each day earlier than before, and finally, are unable to pursue their occupations without pain and without constant demand for rest. If patients do not heed the warning which these symptoms give, the eyes become painful and congested at the slightest effort, and to such a degree often, that sleep does not relieve them. Persistent use of the eyes in this condition is liable to produce symptoms of retinitis, which may terminate in amaurosis. Fortunately the pain becomes so severe as to prevent such use of the eyes, and organic injury of the retina is probably quite a rare result of uncomplicated asthenopia.

This form of the disease has been called accommodative asthenopia, as being dependent upon an abnormal condition of the muscle of accommodation. It is comparatively rare in youth, being observed principally after the twenty-fifth year of age.

The causes of the disease are, chiefly: Undue use of the eyes, especially if there is insufficient light; weakness of the ciliary muscle from general debility, and very often, that condition of the eye described in our last lecture, as Hypermetropia. The hypermetropic eye, even in looking at *distant objects*, is forced to bring the ciliary muscle into a state of partial contraction. In looking upon near objects, it often requires the utmost power of accommodation to render the lens sufficiently convex. The eye while thus expending its total power of adjustment, is in the same relative condition as the muscles of the arm, while supporting the greatest possible weight; the eye, like the arm, becomes more speedily fatigued, and requires rest sooner than when but a part of its power is expended.

The second form of asthenopia depends upon a weakness or insufficiency of one or of both the internal recti muscles, and is therefore termed muscular asthenopia. As you well know, the ciliary muscle and the internal recti act consensually; the ciliary muscle, in contracting, accommodates the eye for near objects, and at the same time the internal recti contract to render the visual axis more convergent. It sometimes happens that the internal recti become weakened or insufficient. They possess sufficient force to rotate the eyes normally without difficulty, and even to retain the axis quite convergent for some time. But when it is required to fix the eyes long upon minute and near objects, the energy of one or both of the muscles is found wanting; there is a sense of fatigue, which speedily in continued effort becomes painful; the muscle loses its power of contraction, and no longer holds the axis of the eye upon the object; its antagonist gains the control, and rotates the eye outwards. The result of this is indistinct vision produced by double images. These two features, double images and temporary external strabismus, distinguish muscular from accommodative asthenopia. It should be observed that the double vision is not usually very marked, but is characterized by a peculiar indistinctness of objects, caused by unsteadiness of the internal rectus, slightly rotating the eye alternately inwards and outwards. Hence, in cases of slight insufficiency, the images are not fully separated, but simply overlap each other to a greater or less extent.

The diagnosis of this form of the disease rests upon the two following means of examination: If the patient is directed to fix the eye upon some minute and near object, simple inspection of the eye affected will often show any divergency which may exist in the axes of the eye. A more delicate means is found in the use of the prism. You are aware that rays of light, on passing through a prism, are bent in the direction of its base. Now, if a prism be held before a normal eye, with the base downwards for instance, the rays of light, on passing through the prism, will be inclined downward, and, consequently, fall upon a portion of the retina lower than the images on the other retina. In this eye, therefore, objects all appear in the upper portion of the field of vision. Hence, you should impress this rule upon your minds: that the images of objects seen through a prism, are formed upon that portion of the retina corresponding to the side of the eye to which the base of the prism is directed. This, however, causes the object to appear in the opposite direction, or in that to which the angle of the prism is placed—or in other words, an object seen through a prism appears above, below, at either side, or in any intermediate direction, according to the direction in which the angle of the prism is held.

Now, if a prism with its base *downwards*, be placed before an eye, in which there is even a very slight insufficiency of the internal rectus, the rays will be bent downwards, while the objects will apparently be thrown, for an instant, directly upwards. Hence, there will be double images, that of the eye with the prism being thrown, for an instant, directly above that of the other eye. This double vision so disturbs the harmony of the action of the muscles of the eyes in their efforts to see single, that almost the slightest preponderance of one muscle readily controls the eye. In this case the external rectus being a little stronger than the internal, at once rotates the eye outward, very much quicker and to a greater extent, than when the prism is not used. Since the prism tends to throw one image directly above that of the other eye, the rotation of the eye outwards carries this second image to the opposite direction, that is to the left, when the right eye is affected, and to the right when the left one is affected. In this way we see plainly the rotation of the eye outwards. Of the farther use of prisms in diagnosis, and of the mode of measuring the insufficiency of a muscle by the number of degrees in the triangle of a prism, which is required to unite double images, we shall speak in a future lecture.

The etiology of muscular asthenopia is sometimes obscure. It is often dependent upon debilitating diseases, in which there is a want of innervation of the general muscular system. It is worthy of notice, that if the eyes are too severely taxed in this condition, the internal rectus may not recover its energy for a very long period.

Acquired short-sightedness and hypermetropia are often causes of muscular asthenopia. If congenital, they are less liable to produce it, since the constant exercise of the internal recti from infancy gives them such strength, that they are able to hold the visual axes more convergent than in the normal eye. After adult age it seems difficult for the internal recti to develop by exercise more force. Hence, in acquired short-sightedness or hypermetropia, one or both of the internal recti are found too weak to produce the necessary convergency. After short and ineffectual efforts to accomplish this, the weaker soon yields to its antagonist, and the eye rotates outward. Rest, application of either tepid or cold water, and looking at a distance, enables the muscle to recover its energy temporarily.

The third form of this disease, or retinal asthenopia, depends upon an obscure pathological condition of the retina. There is a peculiar sensitiveness or irritability of the nerve which forces the patient to avoid certain colors and unsteady lights, especially artificial. Such patients suffer great pain in endeavoring to look long npon any object either near or distant. They cannot pursue the ordinary occupations of life. This condition usually exists to a greater or less extent, as a result of the other forms of asthenopia, as also independent of them. There is much obscurity in the causes of this affection, although the intimate relation between the retina and optic nerve with the sympathetic nerve, and with the nerves of sensation and motion, will explain many of the phenomena of the disease.

In regard to the treatment of these diseases, you should remember that it is more satisfactory in the earlier stages than after the affection has been long established. If there appear the slightest symptoms of the disease, great care is necessary in regard to quantity and quality of light, and kind and duration of labor. Patients, during convalescence after severe, debilitating diseases, in which the general muscular system fails in innervation, should use their eyes on near objects as little as possible.

Short-sightedness of marked degree requires suitable concave lenses, even in reading. Hypermetropia requires double convex lenses. In either of these conditions of the eye, if hyperæsthesia of the retina already exists, all use of the eyes, with scarcely an exception, even with lenses, should be avoided.

In cases of insufficiency of the internal rectus, the use of a suitable prism, with the angles directed outwards, will often relieve the indistinctness of vision. Since the rotation of the eye outwards in these cases, carries the central portion of the retina towards the inner angle of the eye, a suitable prism with the base towards the inner angle of the eye, bends the rays of light towards its base ; consequently, these rays will form images upon the central portion of the retina. In this way prisms overcome double vision, when the visual axes are not both directed upon the same point.

In extreme cases the division of the tendon of the external rectus restores the equilibrium between this muscle and the internal rectus.

Galvanism will sometimes restore the ciliary and internal recti muscles to their normal tone.

Patients are not unfrequently enabled to read and work upon minute objects comfortably by covering the affected eye.

Cold douches to the closed lids, the external application of aconite, morphine, iodine, chloroform around the eyes, are sometimes found to relieve temporarily the pain. To alleviate the photophobia, plain glasses of London smoke, or cobalt blue color, should be worn, as circumstances require.

During the whole treatment, great care should be directed to the condition of the abdominal organs. The thoughts of patients should be diverted from their disease. They should be employed as much as possible in the open air, in travel, and such occupations as will not demand much exercise either of the ciliary or internal recti muscles.

Patients should be assured that there is no danger of "amaurosis," when the eyes are not abused.

When all the precautions have been observed, as above recommended, there may be benefit in exercising the eyes by reading a short time daily, and thus gradually restoring the muscles to strength, and overcoming the peculiar irritable condition of the retina.

Although a careful study of each case will enable you often to relieve the affection, you should remember that there are patients whose condition seems to defy the most skilful treatment.

